# TRPM channels in human cancers: regulatory mechanism and therapeutic prospects

**DOI:** 10.1186/s40364-024-00699-2

**Published:** 2024-12-04

**Authors:** Qinfeng Liu, Mengyu Hu, Shi Li, Xin Zhang, Rui Zhang, Hao Lyu, Shuai Xiao, Dong Guo, Xing-Zhen Chen, Jingfeng Tang, Cefan Zhou

**Affiliations:** 1 National “111” Center for Cellular Regulation and Molecular Pharmaceutics, Key Laboratory of Fermentation Engineering (Ministry of Education), Cooperative Innovation Center of Industrial Fermentation (Ministry of Education & Hubei Province), Hubei Key Laboratory of Industrial Microbiology, Hubei University of Technology, Wuhan, 430074 China; 2 Membrane Protein Disease Research Group, Department of Physiology, Faculty of Medicine and Dentistry, University of Alberta, Edmonton, AB T6G 2R3 Canada

**Keywords:** TRPM channels, Cancer progression, Tumor microenvironment, Signaling pathway, Autophagy, Targeted tumor therapy

## Abstract

The transient receptor potential melastatin (TRPM) channel family has been previously implicated in various diseases, including those related to temperature sensing, cardiovascular health, and neurodegeneration. Nowadays, increasing evidence indicates that TRPM family members also play significant roles in various types of cancers, exhibiting both pro- and anti-tumorigenic functions. They are involved in tumor cell proliferation, survival, invasion, and metastasis, serving as potential diagnostic and prognostic biomarkers for cancer. This paper begins by describing the structure and physiological functions of the TRPM family members. It then outlines their roles in several common malignancies, including pancreatic, prostate, colorectal, breast, brain cancer, and melanoma. Subsequently, we focused on investigating the specific mechanisms by which TRPM family members are involved in tumorigenesis and development from both the tumor microenvironment (TME) and intracellular signaling. TRPM channels not only transmit signals from the TME to regulate tumor cell functions, but also mediate extracellular matrix remodeling, which is conducive to the malignant transformation of tumor cells. Importantly, TRPM channels depend on the regulation of the inflow of various ions in cells, and participate in key signaling pathways involved in tumor progression, such as Wnt/β-catenin, MAPK, PI3K/AKT, p53, and autophagy. Finally, we summarize the current strategies and challenges of targeting TRPM channels in tumor treatment, and discuss the feasibility of combining targeted TRPM channel drugs with cancer immunotherapy.

## Introduction

Transient receptor potential (TRP) channels are a significant family of non-selective ion channels that initiate specific intracellular cascade reactions by regulating ion influx in response to various extracellular stimuli, including thermal, chemical, and mechanical signals [1]. Based on sequence homology, the TRP channel superfamily is further classified into seven subfamilies: TRPV (vanilloid), TRPA (ankyrin), TRPC (canonical), TRPM (melastatin), TRPML (mucolipin), TRPN (NOMPC), and TRPP (polycystin) [2]. As the largest and most diverse subfamily of TRP superfamily, TRPM channels play a crucial role in various physiological processes in the body, such as temperature sensing, redox sensing, and inflammation [3].

**Fig. 1:**
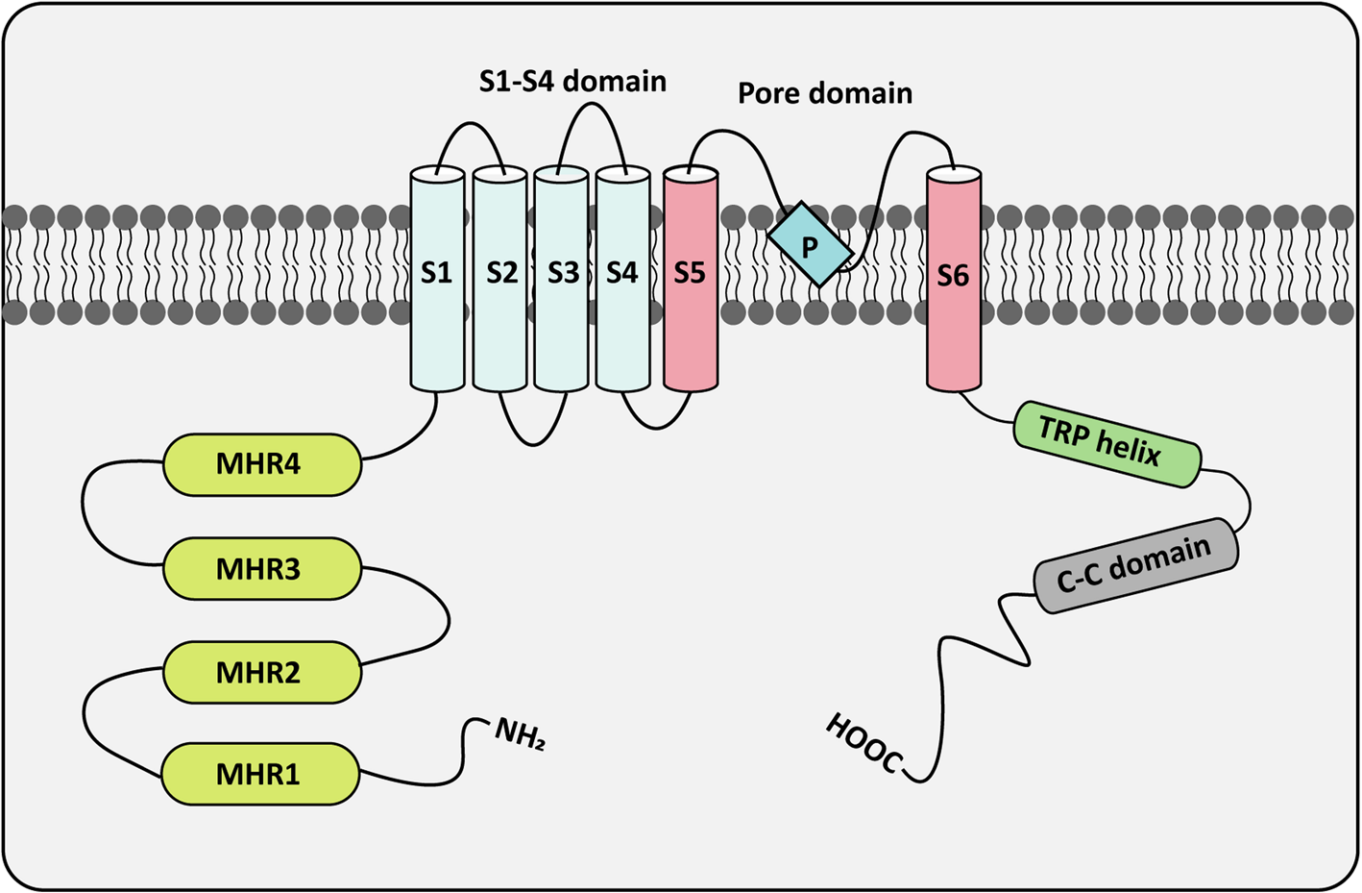
The structure of TRPM channels. The N-terminal region consists of four structural domains called the melastatin homology region (MHR). These domains form pockets that participate in channel formation and the perception of external stimuli [15]. The transmembrane domain (TMD) contains six transmembrane helices (S1-S6). The S4 helix functions as a voltage sensing domain associated with channel activation, while the P-loop between S5 and S6 acts as the ion conduction pore [16]. The C-terminal region comprises a highly conserved TRP helical domain and a coiled-coil domain implicated in the formation of polymer complexes [17]

The TRPM family consists of eight members (TRPM1-TRPM8). Each member has similar structural characteristics and mainly contains three regions: the N-terminal, the transmembrane region, and the C-terminal [4] (Fig. 1). The TRPM family is regulated by various stimulating factors, such as voltage, temperature, and other ligands. Each member of the TRPM family serves various physiological functions, and their aberrant expression is linked to a range of diseases [5] (Table 1). The channel function of TRPM1 is vital for normal melanocyte pigmentation, making it a potential target for addressing pigmentation disorders [6]. TRPM2 is highly expressed in various tissues with high energy demands, where it protects cells from oxidative stress and ischemic injury by regulating calcium absorption, mitochondrial function, and reactive oxygen species (ROS) levels [7]. TRPM6 and TRPM7 are essential for maintaining magnesium homeostasis in organisms. While TRPM6 has limited expression, TRPM7 is widely expressed in various tissues and contributes to cell growth, development, and apoptosis [8]. TRPM8 acts as a cold sensor in the organism, being activated by cooling agents and low temperatures [9].

**Table 1:** The physical functions and related diseases of the TRPM channels family

Channel	Expression	Ion selectivity	Physiological functions	Related diseases	Ref.
TRPM1	Brain, eye, and skin	Divalent cations (Ca^2+^ and Zn^2+^)	Promote melanogenesis and adjust iris constriction	Early age myopia and congenital stationary night blindness	[6]
TRPM2	Brain, bone marrow, spleen, heart, and lung	Divalent and monovalent cations (Ca^2+^ and Na^+^)	Modulate immune system, affect insulin secretion, and regulate body temperature	Bipolar disorder, Alzheimer’s disease, Ischemic stroke, and cardiac ischemic/reperfusion	[7, 10]
TRPM3	Kidney, brain, pituitary, pancreas, eye, and heart	Divalent cations (Ca^2+^ and Zn^2+^)	Affect insulin secretion, regulate neurotransmitter release, and adjust iris constriction	Inherited cataract, epilepsy, and intellectual disability	[11]
TRPM4	Various Cells/Organs	Monovalent cations (Na^+^, K^+^, Cs^+^)	Influence cardiac development and remodeling, control Ca^2+^ oscillations in lymphocytes, and maintain vascular tone	Cardiac conduction block and Brugada syndrome	[12]
TRPM5	Intestine, liver, lung, and sparse chemosensory cells	Monovalent cations (Na^+^, K^+^, Cs^+^)	Mediate signaling in taste cells and contribute to insulin secretion	Diabetes and obesity	[13]
TRPM6	Intestinal and renal epithelial cells	Divalent cations (Mg^2+^ and Ca^2+^)	Participate in epithelial magnesium transport and sustain body magnesium homeostasis	Hypomagnesemia	[14]
TRPM7	Various Cells/Organs	Divalent cations (Mg^2+^, Ca^2+^ and Zn^2+^)	Regulate cellular and systemic magnesium homeostasis	Cardiovascular diseases and Neurodegeneration	[8]
TRPM8	Various Cells/Organs	Divalent and monovalent cations (Ca^2+^ and Na^+^)	Act as a cold receptor	Migraine and Dry eye disease	[9]

Ion channels are the third most common pharmacological target class, following receptors and enzymes. Early studies have shown that TRPM channels hold promising potential in treating diseases such as stroke and bipolar disorder [18]. With advancing research, the TRPM family has been found to play a significant role in the processes of autophagy, proliferation, apoptosis, and metastasis in tumor cells. Importantly, a substantial body of evidence suggests that inhibiting TRPM protein expression or altering its channel activity may be an effective strategy for suppressing tumor progression. The current treatment strategy focuses on developing activators and inhibitors that target TRPM channels. However, most studies remain in the preclinical stage due to issues such as low specificity, limited effectiveness, and a propensity to cause adverse reactions. This article reviews the role and regulatory mechanisms of TRPM channels in common cancers, as well as the challenges and potential solutions associated with existing drugs targeting these channels.

### The role of the TRPM family in various cancers

Evidence increasingly shows that the TRPM family is abnormally expressed or dysfunctional in patients with several cancers, including melanoma, pancreatic, prostate, colorectal, breast, and brain cancer. This section explores the roles of TRPM channels in these cancers and summarizes their mutation frequency and the relationship between altered expression and clinicopathological features (Table 2). The mutation frequency of the TRPM family was derived from cBioPortal’s analysis of TCGA pan-cancer atlas [19, 20].

**Table 2:** The role of the TRPM family in various cancer

Types of Cancer	Channel	Mutation frequency	Expression level	Clinicopathological features	Ref.
Melanoma	TRPM1	9%	↓	Increased invasion and metastasis phenotypes	[21]
	TRPM2	15%	↑	Increased metastasis phenotypes	[22]
	TRPM5	5%	↑	Associated with a poor prognosis for patients	[23]
Pancreatic Cancer	TRPM2	3%	↑	Positively correlated with tumor stage, increased proliferative, and invasive phenotypes	[24, 25]
	TRPM7	2%	↑	Increased proliferation, migration and invasion phenotypes	[26, 27]
	TRPM8	1%	↑	Positively correlated with the size and stage of primary tumor Increased migration and invasion phenotypes	[28, 29]
Prostate cancer	TRPM2	1%	↑	Related to tumor grade and enhanced the proliferation phenotype	[30, 31]
	TRPM4	2%	↑	Increased migration and invasion phenotypes	[32]
	TRPM7	1%	↑	Increased migration and invasion phenotypes	[33, 34]
	TRPM8	1%		Remain controversial (possible staging dependency)	[35–38]
Breast cancer	TRPM2	2%	↑	Increased proliferative phenotype	[39]
	TRPM4	1%	↑	Significantly associated with poorer prognosis, as demonstrated by a higher lymph node status and a higher pathologic prognostic stage	[40]
	TRPM7	2%	↑	Increased proliferation, invasion and spread phenotypes	[41–43]
	TRPM8	2%	↑	Closely associated with proliferation parameters and promoted tumor cell migration	[44, 45]
Colorectal cancer	TRPM2	6%	–		
	TRPM4	2%		Remain controversial (possible staging dependency)	[46, 47]
	TRPM6	7%	↓		
	TRPM7	4%	↑	Positively correlated with tumor infiltration, lymph node metastasis, distant metastasis, and the clinical stage of colorectal cancer	[48]
	TRPM8	5%	↑	Positively associated with tumor cell migration and invasive phenotypes, promoting liver metastasis.	[49, 50]
Brain cancer	TRPM2	2%	↑		[51]
	TRPM3	3%	↑	Negatively correlated with the glioma grade	[52]
	TRPM7	1%	↑	Increased proliferation, migration and invasion phenotypes	[53]
	TRPM8	1%	↑	Increased proliferation, migration and invasion phenotypes	[54, 55]

: Up-regulated expression, : Down-regulated expression, : Unknown

#### Melanoma

The best indicator of melanoma risk is the level of melanin synthesis in the skin. TRPM1 expression shows a positive correlation with melanin content [21]. As the first discovered member of the TRPM channels family, TRPM1 has been found to negatively correlate with the aggressiveness of melanoma cells [21]. Through in situ hybridization, Deeds et al. observed TRPM1 mRNA expression in benign nevi, atypical nevi, and melanoma in situ, while metastatic melanoma exhibited minimal expression [56]. In primary melanoma lesions, reduced TRPM1 expression correlates with increased tumor thickness, suggesting its potential as a predictor of human melanoma development in clinical settings [56]. However, subsequent studies have indicated that the tumor inhibitory function of TRPM1 might be mediated by miR-211 within its intron, rather than TRPM1 mRNA itself [57, 58]. Additionally, TRPM1 is associated with melanoma-associated retinopathy, triggered by ON bipolar cells expressing TRPM1 autoantibodies [59].

The presence of splice variants is a distinctive characteristic of TRPM2. In melanoma, two splice variant transcripts, TRPM2-TE and TRPM2-AS, are notably upregulated [60]. Interestingly, the knockout of TRPM2-TE enhances the susceptibility of melanoma cells to apoptosis and necrosis, suggesting a potential role of this variant in tumor survival mechanisms [60]. Recently, McKamey et al. have found that TRPM2 has a unique nuclear localization in melanoma cell lines and demonstrates that antagonizing TRPM2 can induce an anti-tumor effect in melanoma cells [61]. TRPM5 is conducive to the metastasis of mouse melanoma cells [23]. High TRPM5 expression is associated with lower overall survival (OS) in melanoma patients [23]. NRAS is mutated in 15–20% of melanoma patients, leading to persistent downstream activation of the ERK pathway, resulting in uncontrolled cell growth and migration [62]. Next-generation sequencing (NGS) studies suggest a potential association between TRPM6 and NRAS-driven melanoma, but the specific mechanism remains to be clarified [63]. TRPM8 is functionally expressed in human melanoma cells, and its agonist, menthol, exhibits dose-dependent inhibition of melanoma cell viability [64].

#### Pancreatic cancer

Oncogenic mutations in the *KRAS* gene are a hallmark of pancreatic carcinogenesis. These mutations may result in persistent activation of the KRAS protein, causing its dysfunction [65]. TRPM channels influence oncogenic cell transformation by coordinating with the MAPK and PI3K/Akt/mTOR signaling pathways, which are downstream of RAS.

Approximately 85% of pancreatic cancer (PC) are pancreatic ductal adenocarcinomas (PDAC), originating from the malignant transformation of ductal epithelial cells [66]. According to Lin et al., TRPM2 enhances the proliferation and invasion capacity of PC cells, correlating negatively with survival in PDAC patients [24]. Therefore, TRPM2 may be a potential therapeutic target and prognostic marker for PDAC.

The abundant connective tissue matrix in PDAC contributes to chemotherapy resistance and metastasis, complicating treatment [67]. This stromal remodeling is primarily driven by activated pancreatic stellate cells (PSCs), and TRPM7 is considered a marker of PSC activation [68]. During tumor interstitial remodeling, bioactive elastin-derived peptides (EDPs) interact with the membrane receptor ribosomal protein SA (RPSA) to stimulate cancer cell proliferation. Lefebvre et al.. found that pancreatic cancer cells might produce TRPM7 and RPSA complexes in response to EDP release, which could facilitate cell motility [26].

The expression of TRPM8 is up-regulated in PC patients, making it a potential prognostic factor [69]. Silencing TRPM8 inhibits the expression and activity of multiple drug resistance-associated proteins and affects PC cell apoptosis [28]. Furthermore, the phosphorylation level of TRPM8, a TRPM member frequently undergoing post-translational modifications during cancer progression, is closely associated with PC progression. It has been discovered that the phosphorylation of the TRPM8 Y1022 site is mediated by the tyrosine kinase LCK [29]. This phosphorylation increases TRPM8’s binding ability to the 14-3-3ζ protein, enhancing TRPM8 channel function by promoting TRPM8 polymerization. Additionally, phosphorylation of the TRPM8 Y1022 site elevates PC cell proliferation, migration, and invasion, suggesting a possible therapeutic target for PC patients [29].

#### Prostate cancer

The development of prostate cancer (PCa) is controlled by androgens and primarily occurs in the peripheral prostate epithelial cells. Suppression of androgen levels is currently a main treatment for PCa. However, this approach is ineffective for non-androgen-dependent PCa, which is more aggressive and challenging to treat [70].

TRPM2 is markedly up-regulated in PCa tissue samples and its expression increases with the grade of PCa. Recently, it has been shown that TRPM2 inhibits the autophagy process in PCa [30]. The specific mechanism is detailed in the autophagy section. Initially, Orfanelli et al.. have uncovered that the TRPM2-AS serves as both a prognostic indicator and a treatment target for PCa [71]. It’s worth noting that TRPM2-AS is increased in paclitaxel-resistant PCa cells, which strengthens the durability of PCa cells to paclitaxel and promotes cell progression through the miR-497-5p/FOXK1 axis [72].

Family members TRPM4 and TRPM7 both play key roles in the EMT process in PCa cells. According to Sagredo et al.., TRPM4 induces the expression of the epithelial-mesenchymal transition (EMT) transcription factor Snail1, thus elevating the motility and invasion of PCa cells [32]. However, subsequent studies found that small molecule inhibitors, which can partially inhibit TRPM4 channel activity, have limited effects on PCa [73]. Transforming growth factor β (TGFβ) plays a dual role in cancer, both inhibiting early tumor formation and facilitating tumor invasion and metastasis [74]. As reported by Sun et al., TGFβ not only significantly enhanced the expression level of TRPM7, but also induced the EMT process in PCa cells by mediating Mg^2+^ inward flow through TRPM7 [33].

TRPM8 expression is associated with the stage of PCa development, so it is used as a marker of PCa diagnosis and prognosis in clinical practice [75]. The expression of TRPM8 is governed by androgen, which increases in the initial stage of PCa and decreases after anti-androgen therapy [35]. Androgens enhance TRPM8 expression by activating the androgen receptor, which binds to the androgen response element upstream of the *TRPM8* promoter gene [76]. The role of TRPM8 in PCa cells remains controversial. While various TRPM8 blockers have been shown to suppress different stages of PCa cell growth in vitro [36, 37], TRPM8 demonstrated anti-tumor effects in vivo, limiting tumor growth and metastasis in a mouse transplanted tumor model [35, 38]. Subsequently, the new non-channel function of TRPM8 supports this result. TRPM8, a GDI-like protein, interacts with GDP-binding Rap1A, retaining it in the cytoplasm to block the β1-integrin signaling pathway, thereby inhibiting migration and adhesion of vascular endothelial cells and epithelial prostate cancer cells [77, 78]. In addition, changes in androgen levels modulate TRPM8 channel-mediated PCa cell migration. At low androgen concentrations, raised binding of TRPM8 to the androgen receptor within lipid rafts on the plasma membrane decreases TRPM8 function, thereby facilitating PCa cell motility [79]. Given these mechanisms, TRPM8 presents itself as a potential therapeutic target for PCa, with both its antagonists and agonists showing promise, as discussed in the Targeted Therapy section.

#### Breast cancer

Women are more likely than men to suffer from breast cancer, and treatment focus varies depending on the stage of the disease [80]. Many members of TRPM channels are highly expressed in breast cancer, promoting cancer cell migration and invasion by participating in EMT processes. Unlike typical ion channel functions in the plasma membrane, TRPM2 has a novel performance in the nuclear of human breast cancer cells, where it protects genomic DNA integrity [39]. More importantly, Inhibiting TRPM2 can significantly enhance the cytotoxic effects of chemotherapy drugs in triple-negative (TN) and estrogen receptor-positive (ER^+^) breast cancer. This suggests that targeting TRPM2 could potentially enhance the efficacy of chemotherapy in these patients [81]. Interestingly, in both TN and ER^+^ breast cancer cells, the same family member, TRPM4, and its regulatory protein KCTD5 were upregulated and involved in the cell migration process [82]. Gene Set Enrichment Analysis (GSEA) has also indicated a notable association between TRPM4 transcripts and the EMT gene set, suggesting that this protein may promote breast cancer cell spread by inducing the EMT process [40].

TRPM7 and TRPM8 are highly expressed in human breast ductal adenocarcinoma (hBDA) compared to adjacent non-tumor cells, with their expression levels correlating to specific pathological parameters [83]. TRPM7 is overexpressed in poorly differentiated and highly proliferative breast cancer, while TRPM8 is mainly overexpressed in highly differentiated and poorly proliferative breast cancer [83]. This suggests that TRPM7 may serve as a marker for proliferation in poorly differentiated tumors, whereas TRPM8 could be a prognostic marker for well-differentiated tumors. Furthermore, recent findings highlight TRPM7 as a significant regulator of the EMT process in breast cancer. On the one hand, TRPM7 participates in the EMT process by boosting the expression of vimentin and the STAT3 pathway induced by EGF [41]. On the other hand, TRPM7 maintains mesenchymal characteristics in breast tumor cells by promoting the expression of the EMT transcription factor SOX4 (SRY-related HMG-box4) [84]. TRPM7 may promote tumor metastasis by contributing to the EMT process, and this ability has been demonstrated in mouse xenotransplantation models of breast cancer [85]. Inhibitors of TRPM7 have also been shown to synergistically enhance TRAIL-induced apoptosis in triple-negative breast cancer cells, suggesting potential for combination therapy [86]. Similar to TRPM8 expression in PCa cells which is regulated by androgens, TRPM8 expression levels are regulated by estrogens in breast cancer cells and correlate with ER^+^ status [44]. Additionally, TRPM8 is linked to EMT markers such as E-cadherin, fibronectin, and vimentin, which play roles in the migration and invasion of breast cancer cells [44].

#### Colorectal cancer

Colorectal cancer (CRC) is the third most common cancer globally, with risk factors such as poor diet and obesity increasing the likelihood of its development [87]. Kaya et al. have found that the expression of TRPM2 is up-regulated in mouse models of colitis-associated colon cancer. Its activation can induce oxidative stress and apoptosis in colon cancer cells, suggesting that its activator holds therapeutic potential [88]. Despite advances in CRC treatment, survival rates for patients with advanced stages remain low, highlighting the importance of early detection and intervention. It has been discovered that the hypomethylation status of TRPM2-AS1 promoter can be used as a biomarker for CRC detection, which may be helpful for the early detection of CRC in the future [47].

TRPM4 is highly expressed in human colorectal tumor buds, and its expression level was initially thought to be positively correlated with the EMT process and the invasive growth patterns of tumor cells [46]. However, Wang et al. recently identified TRPM4 as a novel methylated tumor suppressor gene that prevents CRC cell migration and invasion through calcium signaling [47]. This discrepancy suggests that the role of TRPM4 in CRC may be stage-dependent. TRPM4 expression is regulated by the tumor suppressor protein p53, and the loss of p53 function in advanced CRC increases TRPM4 expression, which may explain its differing effects in early and late-stage CRC patients [89].

Studies have shown that an appropriate increase in Mg^2+^ intake may reduce the risk of CRC [90]. As Mg^2+^ channels, TRPM6 and TRPM7 play an important role in the emergence of CRC. The mRNA level of TRPM6 is downregulated in CRC tissues, and its protein level is positively linked with the OS of CRC patients [91]. In contrast, TRPM7 is up-regulated in CRC tissues and promotes the proliferation and invasion of CRC cells by facilitating the EMT process [48].The expression levels of TRPM6 and TRPM7 also affect the drug resistance of cancer cells. Compared to doxorubicin-sensitive human colon cancer cells LoVo, drug-resistant LoVo cells usually exhibit higher Mg^2+^ concentration and lower influx capacity [92]. These drug-resistant cells express lower levels of TRPM6 and TRPM7 [92]. The intestine can absorb and transport both Ca^2+^ and Mg^2+^, with Ca^2+^ competitively inhibiting Mg^2+^ absorption and transport, either directly or indirectly. Patients with a missense mutation in the *TRPM7* gene (Thr-1482 to Ile) who consume a diet with a high Ca^2+^/Mg^2+^ ratio are at increased risk of adenocarcinoma and hyperplastic polyps [93].

TRPM8 is highly expressed in CRC and contributes to liver metastasis. It is also significantly associated with poor prognosis in CRC patients [49]. The antagonist cannabinol and the agonist WS12 for TRPM8 can rapidly desensitize and inhibit the growth of colorectal cancer xenografts and the occurrence of chemically induced colon cancer, highlighting the potential of TRPM8 as a CRC drug target [49, 50].

#### Brain cancer

Gliomas are the most common type of primary brain tumor, with glioblastoma (GBM), a malignant glioma, being particularly lethal. GBM has a five-year survival rate of only 6.8% [94]. Its internal heterogeneity contributes to drug resistance, recurrence, and is closely linked to angiogenesis and invasion. Research has shown that GBM cells facilitate invasion by regulating vascular tone through the activation of K^+^ channels in response to Ca^2+^ signaling [95].

In a study by Alptekin et al.., patients with GBM exhibited significantly higher expression levels of TRPM2, TRPM3, TRPM7, and TRPM8, which were strongly correlated with OS rates [51]. Subsequent studies have also confirmed that TRPM channels are involved in glioma progression and may serve as novel therapeutic targets. Specifically, TRPM2 and TRPM3 demonstrate anti-tumor effects in gliomas, while TRPM7 and TRPM8 may play a role in the malignant transformation of gliomas [96].

Initially, Ishii et al.. have discovered that TRPM2 contributes to H_2_O_2_-induced Ca^2+^ increases and promotes GBM cell death, indicating its potential as a therapeutic target [97]. Recent studies have shown that antioxidant selenium (Se), Eicosapentaenoic acid (EPA), and silver nanoparticles (AgNPs) can induce apoptosis by stimulating TRPM2 channel activity, thereby increasing the anticancer effect of chemotherapeutic drugs cisplatin (CiSP) and docetaxel on GBM [98–100]. In contrast to the anti-tumor effect of TRPM2, Bao et al. found that TRPM2-AS was greatly upregulated in glioma patients and promoted glioma cell proliferation, migration, and invasion [101]. MiR-204, located within the intron of the TRPM3 gene, is downregulated in glioma due to hypermethylation of the TRPM3 promoter. It inhibits the stem cell-like phenotype and migration of glioma cells by targeting SOX4 and EphB2, key regulators of migration and stem cell-related traits [52].

TRPM7 channel activity is essential for the growth of glioma cells, and its kinase domain is associated with cell migration and invasion [53]. TRPM7 is intimately linked to multiple signaling pathways in glioma and has potential as a therapeutic target. Treatment with non-specific TRPM7 inhibitors, Xyloketal B and carvacrol, inhibited GBM cell proliferation and migration, accompanied by decreased levels of p-Akt and p-ERK1/2 [102, 103]. These findings suggest that TRPM7-mediated activation of the MAPK/ERK and PI3K/AKT signaling pathways plays a key role in tumorigenesis [102, 103]. Liu et al.. showed that TRPM7 promoted the proliferation and migration of GBM cells by activating JAK2 / STAT3 and Notch signaling pathways [104]. It is worth noting that TRPM7 may be related to the drug resistance of GBM, which induces the increase of cancer stem cell marker ALDH1 activity [104]. Recently, Wan et al.. discovered that TRPM7 can also reduce the expression of the tumor suppressor miR-28-5p, thereby promoting glial cell proliferation and migration by targeting Rap1b signaling [53]. It was initially found that stimulation with the TRPM8 agonist menthol increased Ca^2+^ concentration in GBM cells, and the cell migration ability increased [105]. Subsequent studies have shown that the BK channel plays a key role in this process by sustaining the elevated Ca^2+^ concentration required for menthol to promote GBM cell migration [106]. Recent studies have further confirmed that TRPM8 not only promotes GBM cell migration but also influences cell survival, proliferation, and apoptosis. TRPM8-mediated Ca^2+^ signaling may disrupt the cell cycle through calcium/calmodulin-dependent protein kinase II (CaMKII) and its downstream targets, cdc25C and cdc2 [54]. In addition, TRPM8 is closely associated with the MAPK signaling pathway, upregulating the expression of ERK, Bcl-2, and cyclin D1 in glioma cells, which is beneficial to cell proliferation [55].

## Regulatory mechanism of TRPM channel in tumor progression

### Tumor microenvironment

The tumor microenvironment (TME) is the cellular environment in which tumor or cancer stem cells exist, playing a pivotal role in tumorigenesis, progression, and therapeutic processes [107]. TRPM channels can not only transmit signals from TME to tumor cells, but also mediate extracellular matrix remodeling, which is conducive to the malignant transformation of tumor cells.

#### Transmitting signals from the tumor microenvironment

The intense metabolic demands of tumor cells create a unique tumor microenvironment (TME) characterized by hypoxia, acidity, and elevated levels of ROS. These conditions influence the activity of TRPM channels on tumor cell membranes. For instance, hypoxia can enhance the activity of TRPM2 and TRPM7 channels. Although the effect of low pH on TRPM6 and TRPM7 remains controversial, it inhibits the activity of TRPM2 and TRPM5 channels [108]. More importantly, alterations in TRPM channel activity affect the uptake of ions by cancer cells, thereby regulating important cellular processes. Wang et al. have found that TRPM2 can respond to ROS signals from the TME and mediate Ca^2+^ influx in esophageal squamous cell carcinoma cells, thereby hindering cell proliferation and encouraging apoptosis [109]. Under the treatment of Chl-T, TRPM2 on the cell membrane of melanoma can also increase the activation of two other potassium channels (BK channel and KCa3.1) on the stimulating membrane by mediating intracellular Ca^2+^, which are involved in the progression of melanoma [22]. As an important part of TME, immune cells also play a crucial role in tumor progression and treatment. TRPM2 is activated by H_2_O_2_ secreted by neutrophils and mediates apoptosis by inducing Ca^2+^ influx in tumor cells [110, 111]. Extracellular acidity is one of the hallmarks of solid tumors and is associated with metastatic processes in TME. It has been shown that the channel activity of TRPM5 is affected by acidic extracellular pH [112]. TRPM5 may rely on its ability to transport K^+^ to amplify extracellular acidic signaling and enhance acidic pH-induced MMP-9 expression to promote melanoma lung metastasis [23].

#### Mediating extracellular matrix remodeling

The TME consists of fibroblasts, endothelial cells, immune cells, extracellular matrix (ECM), and various soluble products [107]. The ECM provides physical support for cells in the TME and is vital for cell adhesion and infiltration.

Matrix metalloproteinases (MMPs) induce the breakdown of ECM components, releasing chemokines, growth factors, and pro-angiogenic factors. This process facilitates the malignant transformation of tumor cells across the tissue barrier [113]. Rybarczyk et al. found that Mg^2+^ influx in cells can boost the enzyme activity of TRPM7, encouraging the invasion of PC cells through the Hsp90/uPA/MMP-2 proteolytic pathway [27]. Subsequent studies have shown that TRPM7 operates through a similar regulatory mechanism in lung cancer. Lung cancer patients exhibit abnormal TRPM7 expression, which activates the Hsp90α/uPA/MMP2 signaling pathway and induces pluripotent transcription factors, thereby enhancing the metastatic phenotype of lung cancer [114].

**Fig. 2:**
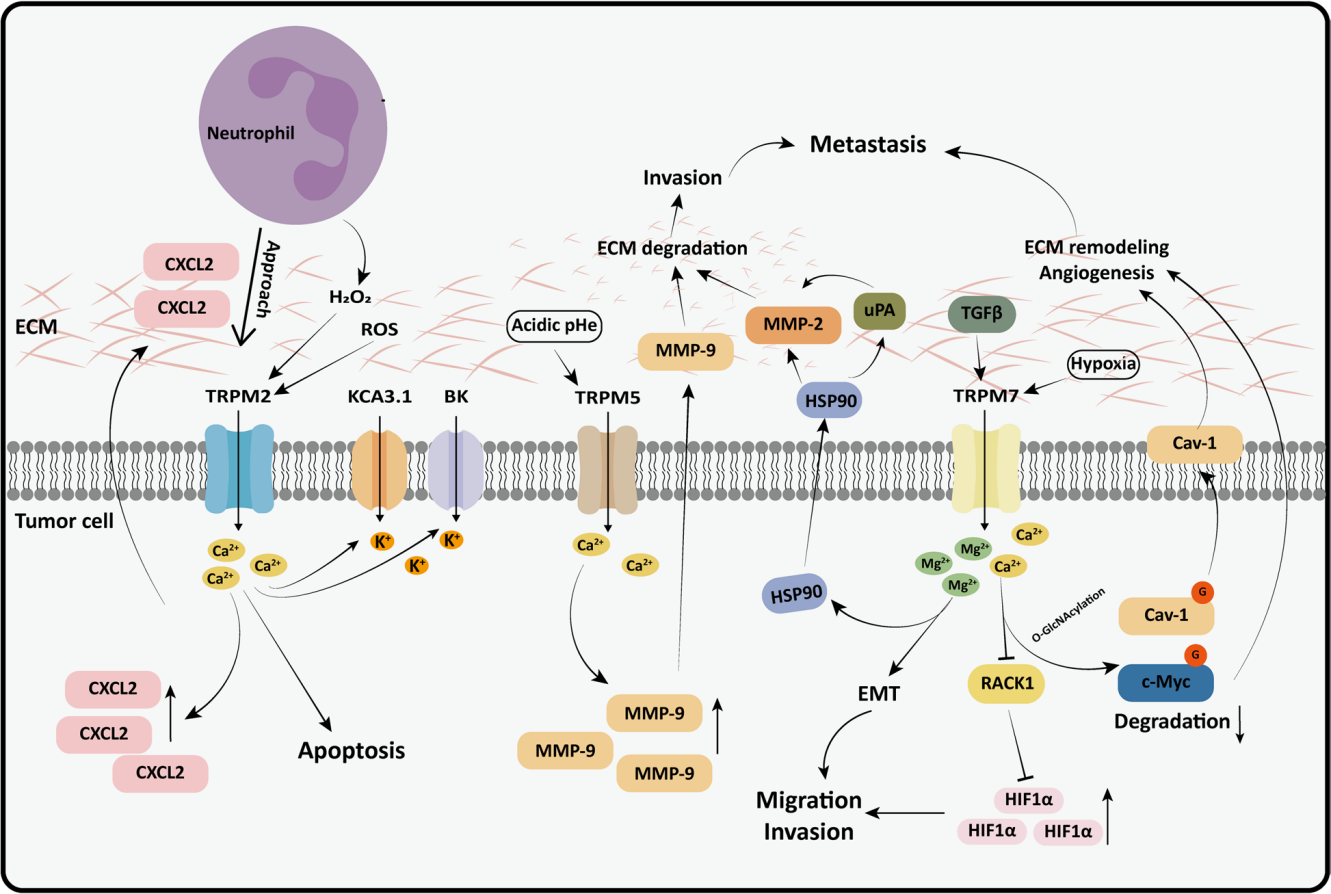
The effect of TRPM channels on tumor microenvironment. TRPM channels in tumor cells can integrate signals from TME and regulate interaction between tumor cells and TME. 1) TRPM2.** a** TRPM2 channels induce Ca^2+^ inward flow to initiate the apoptotic cascade in tumor cells in response to ROS signaling in TME. **b** TRPM2 is activated by H_2_O_2_ secreted by neutrophils within the TME, enabling neutrophils to approach and kill tumor cells by promoting the expression of the chemokine CXCL2. **c** TRPM2 is activated by the oxidizing agent Chl-T and relies on Ca^2+^ influx to stimulate the potassium channels BK and KCa3.1, all of which are jointly involved in melanoma progression. 2) TRPM5. TRPM5 mediates the expression of MMP-9 induced by extracellular acidic PH, facilitating lung metastasis of melanoma. 3) TRPM7.** a** TRPM7 depends on Mg^2+^ inward flow and promotes ECM degradation through the Hsp90α/uPA/MMP2 axis, encouraging cancer cell invasion and metastasis. **b** TGFβ can stimulate the expression of TRPM7 and boost the EMT process of cancer cells through TRPM7-mediated Mg^2+^ influx. **c** TRPM7 inhibits RACK1-mediated degradation of HIF-1α, promoting migration and invasion of cancer cells under hypoxic conditions. **d** TRPM7-dependent calcium signaling enhances the O-GlcNAcylation levels of c-Myc and Cav-1 in cells, preventing their degradation and supporting tumor metastasis

Key regulators of the TME, which are involved in angiogenesis and extracellular matrix remodeling, include caveolin-1 (Cav-1) and c-Myc [115]. It has been reported that TRPM7 relies on calcium signaling to enhance O-GlcNAcylation levels of c-Myc and Cav-1 in cells, inhibiting their degradation and promoting the movement and metastasis of non-small cell lung cancer (NSCLC) cells [116] (Figure 2).

### Signaling pathway

**Fig. 3:**
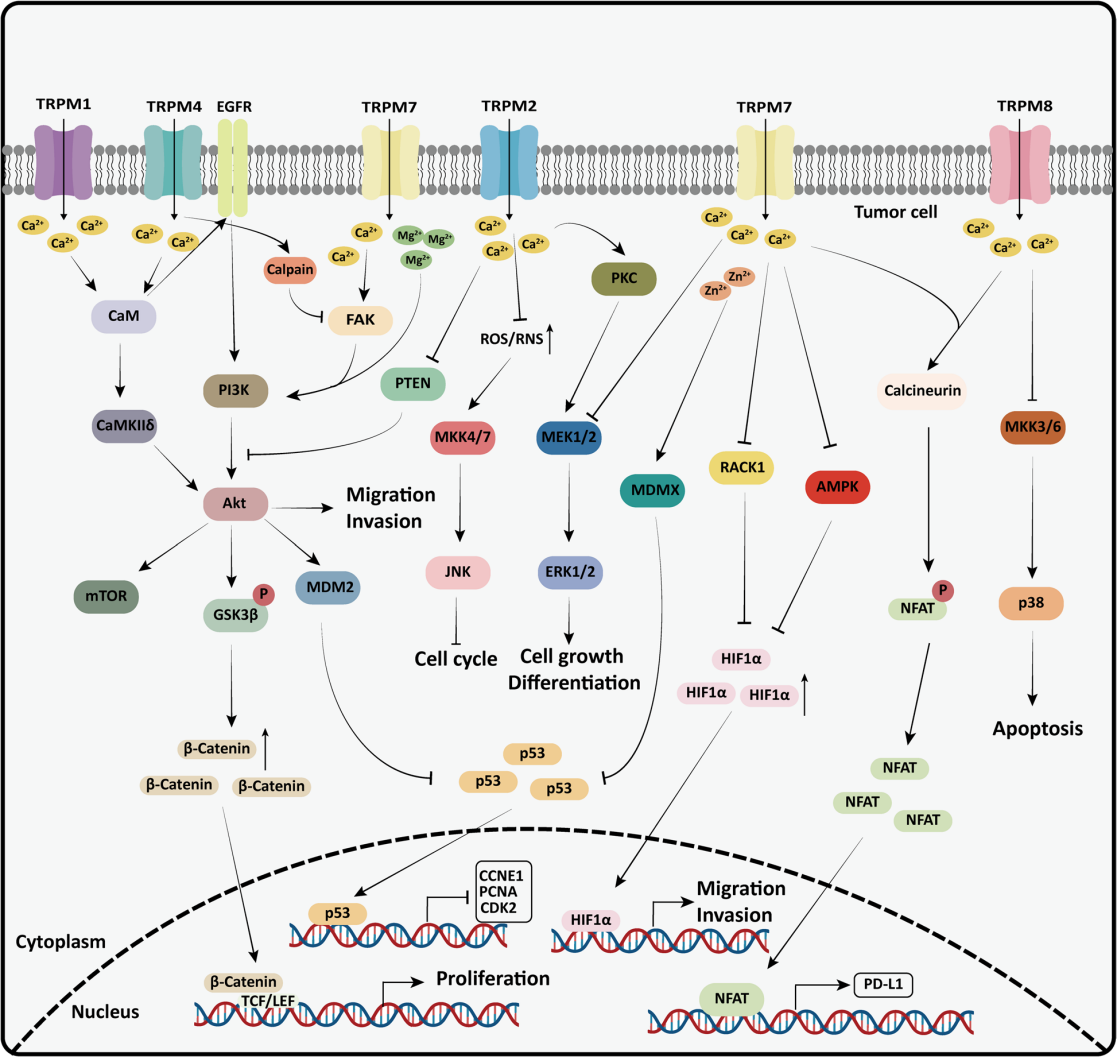
The effect of TRPM channels on signal pathways related to tumor progression. TRPM channels are widely involved in tumor cell proliferation, invasion, and metastasis by regulating signaling pathways related to tumor cell progression. (1) Wnt/β-catenin signaling pathway. Through the Ca^2+^/calmodulin-EGFR signaling axis, TRPM4 controls the activities of AKT1 and GSK-3β, facilitating β-catenin entry into the nucleus. (2) MAPK signaling pathway.** a** TRPM2 activates the MEK pathway by initiating PKC and inhibits JNK activation by reducing ROS and RNS levels in cells. **b** TRPM7 relies on calcium signaling to hinder the ERK1/2 pathway. **c** TRPM8 inhibits JNK and p38 MAPK pathways. 3) PI3k/AKT signaling pathway.** a** TRPM1-dependent calcium signaling activates AKT by rousing CaMKIIδ. **b** TRPM2 inhibits the activity of PTEN, a negative regulator of PI3k/AKT signaling pathway, by mediating calcium influx in cells. **c** TRPM4 upregulates intracellular Ca^2+^ levels to induce calpain-mediated focal adhesion kinase (FAK) hydrolysis, obstructing the PI3K/AKT/mTOR signaling cascade. **d** TRPM7 activates the PI3K/AKT signaling pathway through magnesium and calcium signaling. 4) HIF-1α signaling pathway. In addition to preventing RACK1-mediated HIF-1 degradation, TRPM7 can also slow down HIF-1 degradation by impeding AMPK activity. 5) Calcineurin/NFAT signaling pathway. Both TRPM7 and TRPM8 activate the calcium-regulated phosphatase/NFAT signaling pathway to dephosphorylate NFAT and facilitate its movement into the nucleus. 6) p53 signaling pathway. TRPM7 promotes MDM2-mediated p53 degradation by influencing Mg^2+^ influx, or promotes MDMX-mediated inhibition of p53 transcriptional activity by affecting Zn^2+^ influx

Multiple signaling pathways regulate tumor progression. TRPM channels control intracellular ion influx, and these ions act as second messengers to modulate various signaling pathways. This section examines how TRPM channels regulate key signaling pathways involved in tumor progression, influencing processes such as the cell cycle, EMT, metabolic reprogramming, and immune escape of tumor cells (Fig. 3).

#### Wnt/β-catenin signaling pathway

The Wnt/β-catenin signaling pathway is essential for cancer development. β-catenin, the primary effector of the canonical Wnt pathway, activates the expression of genes related to cell proliferation and invasion once it enters the nucleus [117].

TRPM4 can promote the growth of HeLa cells by activating the Wnt/β-catenin signaling pathway, though its precise mechanism remains unclear [118]. Sagredo et al. recently discovered that TRPM4 may regulate the activity of AKT1 and GSK3β *via* the Ca^2+^/calmodulin-EGFR signaling axis, increasing the transcriptional activity of β-catenin and enhancing the proliferative capacity of PCa cells [119].

Gene enrichment analysis demonstrated that the high expression of TRPM8 is associated with the excessive activation of the Wnt-Frizzled signal and the downregulation of Adenomatous polyposis coli (APC) in colon cancer patients [49]. This suggests that TRPM8 may facilitate tumor growth by enhancing the Wnt/β-catenin signaling pathway. In the non-canonical Wnt pathway, the TRPM8 chaperone protein TCAF2 (TRP channel-associated factor 2) induces Wnt5a secretion in peritumoral cells by preventing the expression and activity of TRPM8 [120]. This process causes the phosphorylation of STAT3 in the tumor cells, thus promoting liver metastasis of colorectal cancer [120].

#### MAPK signaling pathway

The MAPK signaling pathway is involved in cell proliferation, differentiation, and apoptosis [121]. Aberrant activation of several proteins in this pathway is a crucial factor in the genesis of many cancers [121].

There is evidence that the PKC family can prompt the MAPK pathway through multiple mechanisms. Specifically, PKCα, PKCε, and PKCδ support the proliferation and metastasis of PC by activating Raf-1 [122]. TRPM2 can directly activate PKCα by regulating Ca^2+^ levels in PC cells and indirectly activate PKCε and PKCδ by controlling diacylglycerol (DAG) levels, which in turn affects the downstream MAPK/MEK pathway to promote PC growth [25]. In mammalian cells, elevated levels of reactive nitrogen species (RNS) and ROS can activate the JNK signaling pathway [123]. When TRPM2 is downregulated, lung cancer cells accumulate ROS and RNS, leading to DNA damage and cell cycle arrest caused by JNK activation, eventually resulting in cell death [124].

Regulator of G-protein signaling 4 (RGS4) is a regulator of the G protein signaling family, and its expression is suppressed by the JNK/AP1 and PI3K/AKT/GSK3β signaling pathways [125]. It has been reported that TRPM2-AS is significantly elevated in glioma cells and may promote cell proliferation, migration, and invasion through the JNK/c-Jun/RGS4 signaling pathway [101].

TRPM7 silencing is dependent on calcium signaling to activate the ERK1/2 pathway, thereby causing apoptosis in bladder cancer cells [126]. Silencing TRPM8 enhances apoptosis in PCa cells induced by the drug epirubicin through the activation of JNK and p38 MAPK pathways [127].

#### PI3K/AKT signaling pathway

The PI3K/AKT signaling pathway plays a crucial role in regulating phenotypes associated with cancer cell metastasis [128]. Factors such as PTEN (phosphatase and tensin homolog), CaMKIIδ (Ca^2+^/calmodulin-dependent protein kinase IIδ), and focal adhesion kinase (FAK) are upstream regulators of this pathway [128]. Their function is intimately linked to the ability of TRPM channels to control Ca^2+^ influx.

Activated by the intracellular calmodulin (CaM)-Ca^2+^ complex, CaMKIIδ can phosphorylate a wide range of substrates, including AKT. TRPM1 has been demonstrated to increase Ca^2+^ levels in melanoma cells and motivate CaMKII, which in turn activates AKT, accelerating tumor progression [129]. PTEN dephosphorylates PI3P at the cell membrane, antagonizing the kinase activity of PI3K. TRPM2 controls calcium influx in gastric cancer cells, which suppresses the ability of PTEN to negatively regulate the PI3K/AKT signaling pathway, thus fostering the EMT process [130]. The regulatory subunit p85 of PI3K is recruited to the phosphorylation site of FAK, stimulating PI3K activity to activate AKT [131]. TRPM4 prevents the PI3K/AKT/mTOR signaling cascade by triggering calpain-mediated hydrolysis of FAK *via* intracellular calcium signaling, thereby impeding the migration and invasion of CRC cells [47]. Cao et al. have found that knockdown of TRPM7 impairs bladder cancer cell motility and invasion, accompanied by significant downregulation of p-FAK and its downstream p-PI3K and p-AKT [126]. Previous research by Liu et al. has demonstrated that TRPM7 stimulates PI3K/AKT signaling by upregulating Ca^2+^ levels in cells and promotes EMT processes in ovarian cancer cells [132]. Recently, Auwercx et al. have revealed a new regulatory mechanism in which TRPM7 activates the PI3K/AKT pathway in a Mg^2+^ inflow manner, increasing PSC proliferation by altering the expression levels of cell cycle regulators and p53 [68].

GSK3β is a PI3K/AKT downstream regulator, and its sustained activation is associated with tumor cell growth and proliferation [133]. Upregulation of TRPM8 was observed by Liu et al. to greatly increase the phosphorylation of AKT in breast cancer cells, indicating that the AKT/GSK3β signaling pathway was activated to stimulate the progress of EMT in these cells [45]. TRPM8 may also promote the progress of EMT in colon cancer cells through the AKT/GSK3β signaling pathway, facilitating liver metastasis [134].

#### HIF-1α signaling pathway

Hypoxia-inducible factor 1α (HIF-1α), as the most substantial hypoxia response molecule, is elevated in hypoxia and controls the production of genes that respond to hypoxia [135]. However, it is rapidly degraded under normoxic conditions *via* the intracellular oxygen-dependent ubiquitin protease degradation pathway [136].

Hypoxia is the primary pathological factor contributing to castration resistance and metastasis in prostate cancer. The accumulation of HIF-1α is implicated in the EMT process of PCa cells triggered by hypoxia [137]. In patients with androgen-independent PCa, TRPM7 enhances hypoxia-induced malignant migration and invasion of cancer cells by impeding the degradation of HIF-1α mediated by the receptor for Activated C Kinase 1 (RACK1) [138]. Additionally, HIF-1α and AMPK signaling pathways are also involved in glycolysis and oxidative phosphorylation, which are important for the metabolic reprogramming of tumor cells [139]. Cancer cells preferentially utilize the glycolytic pathway to increase ATP production and create an acidic microenvironment favorable for tumor growth, primarily through the production of lactic acid. It has been showed that silencing TRPM7 may activate AMPK, which aids the degradation of HIF-1α ubiquitinated proteasome in ovarian cancer. This allows a shift in the glycolytic process to an oxidative phosphorylation process, hindering the growth of ovarian cancer cells [140]. TRPM7 also relies on its calcium channel function to boost the transcription of glucose transporter GLUT3, thereby driving tumorigenesis and angiogenesis through the glycolysis pathway [141].

#### Calcineurin/NFAT signaling pathway

Translocation activation of the nuclear factor of activated T cells (NFAT) members is widely recognized as an essential factor in the oncogenic transformation of malignant tumors [142]. Calcineurin, which is activated in response to a sustained increase in calcium ions, promotes the nuclear translocation of NFAT by dephosphorylating it. Activated NFAT proteins can conduct the transcriptional activity of proteins involved in cell survival, migration, and angiogenesis by forming complexes with vital oncogenic proteins [143]. According to Chen et al., TRPM7 can stimulate the calcineurin/NFAT signaling pathway, thus favouring the metastasis of head and neck squamous cell carcinoma [144].

Programmed death ligand-1 (PD-L1) causes T-cell depletion and leads to immune escape [145]. Lan et al. have discovered a significant correlation between the expression of PD-L1 and TRPM8, noting that the upstream region of the PD-L1 promoter contains potential binding sites for NFATc3 [146]. TRPM8 promotes the production of PD-L1 through the calcineurin/NFATc3 pathway in esophageal cancer cells, which aids their proliferation and immune escape [146].

#### p53 signaling pathway

p53 serves as a pivotal tumor suppressor in human cancers. In CRC patients, p53 alters Ca^2+^ signaling within cells by suppressing TRPM4 expression [89]. Both zinc excess and deficiency can lead to p53 misfolding and loss of function through various mechanisms [147]. MDM2 and MDMX, crucial negative regulators of p53, are zinc-containing proteins. Wang et al. have found that TRPM7 interacts with MDMX and hinders the degradation of MDMX by mediating intracellular Zn^2+^ influx, thus encouraging the growth and migration of breast cancer cells [42].

#### Autophagy

Autophagy helps maintain cellular homeostasis under stress conditions, thereby enhancing cell viability [148]. During this process, damaged cytoplasmic components are presented to the lysosome for degradation. Macroautophagy, commonly known as autophagy, is the most extensively studied type and comprises four stages: initiation, nucleation, extension, and maturation. Each stage of autophagy relies on proteins encoded by distinct autophagy-related genes (ATGs) [149]. Numerous studies have demonstrated a strong correlation between tumor stage and the impact of autophagy on tumor cells. In the early stage of tumorigenesis, autophagy can prevent DNA damage and arrest tumor formation. However, as tumors progress to advanced stages, autophagy enables tumor cells to resist stress conditions like nutrient deprivation and chemotherapy, thereby promoting tumor progression [150]. A large number of investigations have demonstrated that TRPM channels primarily rely on calcium signaling to control autophagy and regulate tumor cell death and growth through this process [151] (Table 3).

**Table 3:** Mechanisms by which TRPM channel family affects the autophagy process of cancer cells

Channel	Tumor types	Roles in autophagy	Biological effects	Ref.
TRPM2	Neuroblastoma	Compared with TRPM2-S isoform, plasma membrane expression of TRPM2-L promotes Mitophagy by increasing (HIF)−1/2α expression and enhancing BNIP3 content	Reduce oxidative stress injury	[152]
	Gastric cancer	Promote autophagy in a JNK signaling pathway-dependent manner	Maintain mitochondrial function	[153]
	Cervical cancer	Prevent the Beclin1 complex from assembling under oxidative stress	Induce tumor cell mitochondria to break under oxidative stress conditions	[154, 155]
	Prostate cancer	Reduce the transcription level of ATGs and inhibit the autophagy process		[30]
TRPM3	Renal cell carcinomas	Enhance autophagy by encouraging the phosphorylation of AMPK	Conducive to cancer cell growth	[156, 157]
TRPM7	Neuroblastoma	Contribute to phosphorylation of AMPK and promote autophagy		[158]
	Cervical cancer, Pancreatic cancer, Melanoma	Interfere with the binding between STX17 and VAMP8, preventing the mature stage of autophagy	Cause cell cycle arrest and apoptosis	[159]
TRPM8	Breast cancer, Cervical cancer, Colorectal cancer	Interact with AMPK and activate AMPK-ULK1 pathway to facilitate autophagy	Encourage the migration and multiplication of cancer cells	[160]

##### TRPM channels positively regulate autophagy and promote tumor progression

Autophagy, an essential antioxidant pathway in cells, is able to alleviate the damage caused by oxidative stress. Mitophagy reduces the production of ROS in cells by removing damaged mitochondria [161]. TRPM2 channels and their isoforms coordinate both autophagy and oxidative stress processes in neuroblastoma. Full-length TRPM2-L can up-regulate the expression of forkhead box transcription factor 3a (FOXO3a) and superoxide dismutase 2 to alleviate the effect of oxidative stress on neuroblastoma. In contrast, cells expressing TRPM2-S subtypes lack this function [162].

Compared with TRPM2-S, TRPM2-L increases Ca^2+^ entry and mitochondrial Ca^2+^ uptake in neuroblastoma under oxidative stress [152]. Increased intracellular Ca^2+^ promotes mitophagy by enhancing the expression of hypoxia-inducible factor (HIF)−1/2α and the mitochondrial autophagy receptor BNIP3, which leads to decreased ROS levels and facilitates tumor growth [152]. Almasi et al. have reported that TRPM2 can increase the expression of *BNIP3* and *ATGs* in gastric cancer cells in a JNK-dependent manner, boosting autophagy/mitophagy and enhancing cancer cell survival [153].

Autophagy activity and the expression of autophagy regulator LC3B are associated with the growth of clear cell renal cell carcinoma (ccRCC), which is characterized by the absence of von Hippel-Lindau tumor suppressor (VHL) in its early stages. It has been shown that VHL induces transcription of intron 6 of the TRPM3 gene, and its product MiR-204 inhibits LC3B expression [156]. Hall et al. have discovered that TRPM3 stimulates LC3A/B-mediated autophagy, which aided in the proliferation of ccRCC [157]. In ccRCC, TRPM3 contributes to autophagy by regulating Ca^2+^ influx, supporting the activation of calcium/calmodulin-dependent protein kinase kinase 2 (CaMKK2) and the phosphorylation of AMPK [157]. Similarly, TRPM7 can enhance basal autophagy levels in SH-SY7Y cells through a related mechanism [158]. Recent studies in our laboratory have revealed that TRPM8 is upregulated in various cancers with high levels of autophagy. TRPM8 predominantly interacts with AMPK, activating it, which encourages ULK1 phosphorylation and increases basal autophagy [160]. Additionally, TRPM8 can improve the migration and proliferation of breast cancer cells by encouraging autophagy.

##### TRPM channels negatively regulate autophagy and inhibit tumor progression

**Fig. 4:**
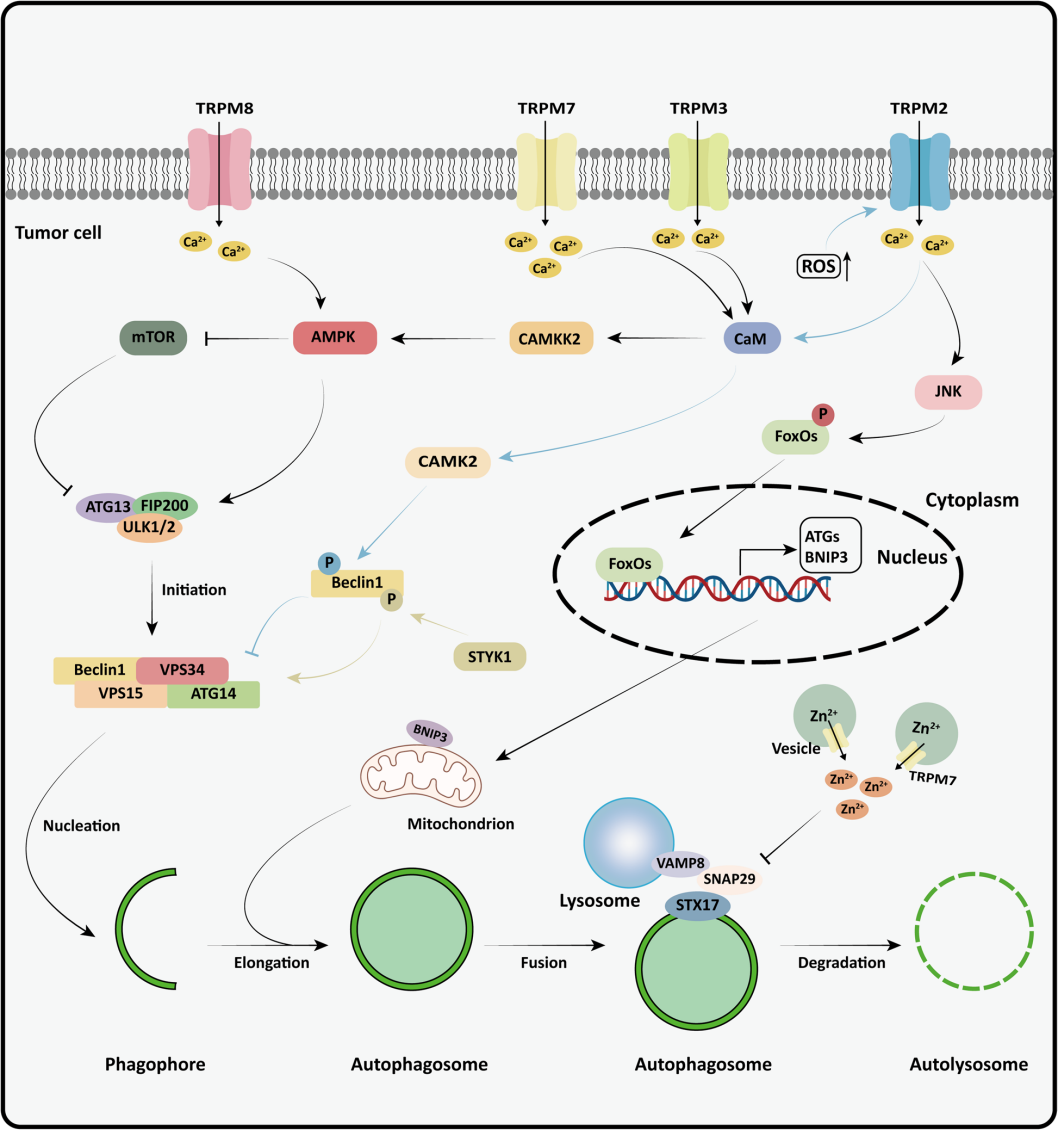
The effect of TRPM channels on autophagy in tumor cells. The effect of TRPM channels on the autophagy process in tumor cells varies by cell type and status. 1) TRPM2.** a** Under oxidative stress, TRPM2 activates CAMK2 by Ca^2+^ influx, inhibiting VPS34 complex binding by phosphorylating Beclin1, which hinders autophagy (marked by blue arrows). **b** TRPM2 enhances the expression of BNIP3 and ATGs in a JNK-dependent manner, promoting autophagy. 2) TRPM3. TRPM3 activates CaMKK2 to promote AMPK activation in a Ca^2+^-dependent manner, supporting autophagy. 3) TRPM7.** a** Similar to TRPM3, TRPM7 boosts autophagy by activating CAMKK2. **b** TRPM7 inhibits the fusion of autophagosomes and lysosomes by releasing Zn^2+^ in vesicles. 4) TRPM8. TRPM8 contributes to autophagy by activating AMPK

TRPM2 has deficient expression and impaired function in various cancer cells, which may help these cells escape oxidative stress-induced cell death [154]. TRPM2 relies on Ca^2+^ inward flow to activate CAMKII, which encourages the dissociation of Beclin1-VPS34 complex by phosphorylating Beclin1, thus preventing autophagy and inducing cell death [154]. Wang et al. also showed that the TRPM2-CaMKII cascade activated by oxidative stress induces ROS production in tumor cells, ultimately resulting in mitochondrial fragmentation and cell death [155]. Tektemur et al. have demonstrated that interfering with TRPM2 enhances the transcriptional levels of *ATGs* and promotes autophagy in PCa cells [30]. The vast majority of TRPM7 is localized to vesicle membranes, which store large amounts of zinc in the cell and regulates the Zn^2+^ content [159]. Xing et al. have revealed that TRPM7 interferes with the interaction between STX17 on the autophagosome and VAMP8 on the lysosome in the cytoplasm by releasing Zn^2+^ from the vesicles, thereby disrupting autolysosome formation and inhibiting the autophagy maturation stage [159]. The further induction of apoptosis, cell cycle arrest, and increased ROS by TRPM7-mediated restriction of autophagy prevents tumor development and metastasis (Figure 4).

## Potential approaches to target TRPM channels for tumor treatment

Many studies have shown that targeting TRPM channels is an effective strategy for tumor treatment. Regulating TRPM channel activity reduces cancer cell proliferation, migration, and invasion, thereby impeding tumor growth and spread. Furthermore, controlling TRPM channel activity can lower the risk of cancer cells developing drug resistance and improve the chemotherapeutic efficacy of particular anticancer medications. Over recent decades, efforts have been made to develop agonists and inhibitors that modulate TRPM channel activity, but progress has been slow. To date, no TRPM modulators for cancer treatment have been approved by the FDA. This section reviews the current research progress and challenges surrounding TRPM-targeted drugs, and explores the potential of combining TRPM-targeted therapies with cancer immunotherapy.

### Current therapeutic strategies—TRPM channel activators/inhibitors

In recent years, numerous TRPM channel regulators that can suppress tumor progression have been identified (as shown in Table 4). These regulators influence apoptosis, autophagy, proliferation, and metastasis in cancer cells by targeting TRPM channels, offering a potential strategy for cancer treatment. In this section, we discuss current areas of contention, as well as the latest discoveries.

**Table 4:** The modulators of TRPM channel that inhibit tumor progression

Target	Medicines	Tumor types	Related mechanism	Ref.
TRPM2	8-Br-ADPR/-	Prostate cancer	Block apoptosis and activate autophagy, resulting in a significant decrease in the viability of PC-3 cells.	[30]
	2-APB/-	Breast cancer	Accelerate DNA damage in cancer cells and reduce cell proliferation levels	[39]
	Clotrimazole/-	Melanoma	Reduce cancer cell proliferation	[61]
	Eicosapentaenoic acid/+	Glioblastoma	Enhance cisplatin-induced oxidative stress and apoptosis in cancer cells	[99]
TRPM3	MFA/-	Clear cell renal cell carcinoma	Inhibit autophagy process in cancer cells	[157]
TRPM4	NBA/-	Colorectal cancer	Hinder the growth and viability of cancer cells	[163]
TRPM5	TPPO/-	Melanoma	Lessen extracellular acidic PH-induced MMP-9 production and spontaneous metastasis in melanoma	[23]
TRPM7	Carvacrol/-	Glioblastoma	Block cancer cells from proliferating and migrating, and trigger apoptosis	[164, 165]
	Waixenicina/-	Lung cancer	Suppress Hsp90α/uPA/MMP2 signaling to prevent cancer cells from exhibiting the characteristics of cancer stem cells	[114]
	Clozapine or Naltriben/+	Pancreatic cancer	Inhibit autophagy to suppress tumor growth and metastasis in vivo	[159]
TRPM8	AMTB and JNJ41876666/-	Prostate cancer	Reduce the proliferation rate of tumor cells	[36]
	BCTC and Sesamin/-	Prostate cancer	Interrupt cell cycle progression in cancer cells	[37, 166]
	D-3263/+	Prostate cancer	Facilitate TRPM8-driven calcium cytotoxicity	[167]
	AMTB/-	Osteosarcoma	Exert anti-tumor effects by inhibiting TGFβ signaling	[168]
	AMTB/-	Melanoma	Enhance G2/M phase arrest induced by γ-irradiation in tumor cells	[169]
	WS12/+	Colorectal cancer	Hamper the growth and genesis of tumors by interfering with the Wnt signaling pathway	[49]
	Cannabigerol/-	Colorectal cancer	Promote the apoptosis of cancer cells and inhibit their growth	[50]

- : Inhibitor, + : Activator

The role of TRPM2 in mediating oxidative stress-induced cell death remains controversial. Early studies suggest that TRPM2 promotes oxidative stress-induced cancer cell death, while other research shows it can enhance cancer cell survival under oxidative stress conditions [154, 155, 170]. On the one hand, activating the TRPM2 channel is being considered as a potential treatment strategy for GBM. Se, EPA, and AgNPs, when combined with the CiSP and docetaxel, induce apoptosis by triggering mitochondrial oxidative stress to activate the TRPM2 channel to increase Ca^2+^ influx [98–100]. On the other hand, specific inhibitors targeting TRPM2 have great clinical potential. Recently, Chen et al. showed that inhibiting TRPM2 helps prevent and overcome acquired resistance to third-generation EGFR-tyrosine kinase inhibitors (such as osimertinib) in patients with EGFR-mutant lung cancer [171]. Osimertinib can inhibit the expression of TRPM2 through a Vitamin D Receptor-dependent (VDR) mechanism, thereby inducing ROS generation, DNA damage, and apoptosis in NSCLC cells [171]. Moreover, specific knockdown of TRPM2 using RNAi silencing or shRNA lentivirus technology can significantly enhance the therapeutic effect of various anticancer drugs (such as paclitaxel, doxorubicin, and azithromycin) [81, 153]. Mechanistically, the transcriptional regulators FOXM1 and E2F1 were expressed at lower levels when TRPM2 was downregulated, which hindered the development cycle of cancer cells and increased their susceptibility to azithromycin treatment [172].

Many studies have shown that reducing the expression and activity of TRPM7 can be used as a method to inhibit a variety of tumor progression. As a specific antagonist of TRPM7, waixenicin A not only exhibits a unique inhibitory mechanism but also reduces the expression of endogenous TRPM7 [173]. Treatment with waixenicin A significantly reduced the viability of GBM cells and inhibited tumor sphere formation in lung cancer cells [114, 173]. In addition, high expression of TRPM7 in patients with head and neck squamous carcinoma was associated with resistance to CiSP, and shRNA-mediated silencing of TRPM7 significantly inhibited cancer cell colony formation and tumor ball formation, thereby synergistically enhancing the therapeutic efficacy of CiSP in this cancer type [144]. Recently, Xing et al.. found that TRPM7 inhibits autophagy in cancer cells, and treatment with TRPM7 chemical agonists (clozapine or naltriben) suppresses tumor growth and metastasis in vivo [159].

As discussed in the prostate cancer section, TRPM8 is a promising target for treatment. In cellular experiments, some antagonists targeting TRPM8, AMTB, JNJ41876666, BCTC, and sesamin, showed varying degrees of inhibitory effects on PCa cells, which involved both androgen-dependent and non-androgen-dependent PCa cells [36, 37, 166]. In the orthotopic xenograft mouse model, lipid nanocapsule-encapsulated TRPM8 agonist WS-12 can effectively reduce the metastasis of androgen-independent prostate cancer PC-3 cells that stably expressing TRPM8 [38]. Notably, a phase I clinical trial conducted in 2009 tested the effects of the TRPM8 agonist D-3262 on solid tumors [174]. Although early study suggested that individuals with advanced prostate cancer had stable disease while using the medication, there are no updated clinical findings regarding the treatment. Recently, Genovesi et al. found that D-3263 significantly enhanced the pro-apoptotic activity of enzalutamide and docetaxel in invasive mouse prostate cancer cells [167]. This suggests that targeting TRPM8 has the potential to fill a crucial gap in clinical therapies for metastatic castration-resistant prostate cancer. Targeting TRPM8 can also be combined with certain anticancer drugs. Knockdown of TRPM8 enhances the chemosensitivity of epirubicin in PCa cells and reduces gemcitabine resistance by modulating gemcitabine uptake and accumulation [28, 127]. However, existing studies have shown that the use of TRPM8 channel modulators may cause side effects, including thermoregulation and temperature sensory disorders. Recently, Aierken et al. have developed an activation mode cyclic peptide inhibitor DeC-1.2, which hinders the ligand activation of TRPM8 without affecting its cold activation, using the hot spot center calculation design strategy based on the TRPM8 structure. This inhibitor provides effective analgesia for Oxaliplatin-induced cold hypersensitivity pain without affecting the body temperature of the animals [175]. This study offers valuable insights for future drug design targeting TRPM8 channels.

### Potential future approaches—targeting TRPM channel combined with cancer immunotherapy

Cancer immunotherapy is an ideal method for manipulating the immune system to suppress and kill tumor cells [176]. The combination of targeted therapy and immunotherapy is becoming an important strategy in cancer treatment. Recent studies have indicated that TRPM channels play a role in the infiltration of various immune cells and help reduce immunosuppressive effects within the TME. Therefore, drugs targeting TRPM channels hold potential for use in combination with cancer immunotherapy.

Numerous types of tumor-associated immune cells reside in the TME, each with either antagonistic or promoting functions towards tumors. Understanding the role of TRPM channels in innate and adaptive immune cells will aid in developing advanced treatment strategies. Natural killer cells (NK) are prototypical innate lymphocytes capable of directly eliminating tumor cells *via* cytolytic granules [177]. Additionally, they collaborate with other immune cells to participate in immune responses through pro-inflammatory cytokines and chemokines. TRPM8 RNA released by normal cells and prostate cancer cells is endocytosed by epithelial cancer cells, which accelerates aseptic inflammation in the prostate cancer microenvironment by activating TLR3-NF-kB/IRF3 pathway, promoting NK cell infiltration and anticancer activity [178]. Neutrophils, the most prevalent form of white blood cell in the body, have a dual function in cancer. Recent studies indicate that neutrophils can play a crucial role in anti-tumor immunity, where they are activated by T-cells and help to kill tumor cells that T-cells are unable to eradicate [179]. H_2_O_2_ released by neutrophils in the TME activates TRPM2, causing Ca^2+^ to enter tumor cells and initiate the apoptotic cascade response [110, 111]. During the epithelial-mesenchymal transition (EMT), the upregulation of TRPM2 expression in tumor cells is linked to an increase in CXCL2 secretion, indicating that TRPM2 is also responsible for maintaining neutrophil recruitment [180]. T cells, essential for adaptive immunity due to their potent tumor-killing capacity and specificity in recognizing antigens, are a fundamental element of immunotherapy. Recent studies suggest that TRPM2 may be involved in the activation process of T cells and serve as a marker associated with immune infiltration in clear cell renal cell carcinoma [181, 182]. Furthermore, it has been discovered that the expression of TRPM2 in ovarian cancer is positively linked with a range of immune cells and immunological checkpoints, including ICOSLG and CD40 [183]. These findings illustrate the potential value of drugs targeting TRPM2 in combination with cancer immunotherapy.

In recent years, a spectrum of immunotherapies, such as immune checkpoint inhibitors (ICIs), cancer vaccines, and adoptive cell transfer (ACT), has demonstrated remarkable advancements. TRPM8 has been used as a potential target for dendritic cell (DC) vaccines for prostate cancer. Mature DCs produced from monocytes of PCa patients can induce specific CD8^+^ cytotoxic T-cells (CTLs) response in vitro after loading TRPM8-derived peptides. However, in phase I clinical trials, reactive expansion of CD8^+^ T cells to TRPM8-derived peptides was not detected in patients treated with DC vaccines [184]. Antagonistic PD-1/PD-L1 monoclonal antibodies, as prominent agents within ICIs, have achieved significant success in the past decade. However, the majority of patients in clinical settings still do not benefit from them, largely due to the emergence of drug resistance. Emerging evidence indicates that specific small molecule drugs, which exert dual inhibitory effects on key oncogenic signaling pathways and PD-L1 expression in tumor cells, hold promise when combined with existing ICIs [185]. As outlined in the signaling pathways section, TRPM8 plays a crucial role in oncogenic pathways such as Wnt/β-catenin, MAPK, and PI3K/AKT. Moreover, TRPM8 has been implicated in promoting PD-L1 expression in esophageal cancer cells [146]. This suggests that small molecule drugs targeting TRPM8 may offer potential in future combinations with ICIs.

Necrosis-inducing cancer therapies typically strongly activate immune cells and cause ICD (immunogenic cell death). Recent studies have revealed that necrosis-inducing anticancer therapies targeting TRPM4, in combination with immunotherapeutic agents, could be effective for treating the majority of solid tumors that do not display neoantigens. TRPM4 plays a critical role in cell death and immune activation following treatment with various necrosis-inducing anticancer therapies, such as BHPI, ErSO, LTX-315, Englerin A, calcium electroporation, and Aprepitant. These therapies usually induce an increase in Ca^2+^ in cells, triggering calmodulin to open the Na^+^ channel TRPM4. This leads to cell expansion, rupture, and the infiltration of significant amounts of extracellular Na^+^ and water, stimulating immune cells to eliminate dead cellular remnants [186].

Even though inhibitors and activators targeting TRPM channels have shown encouraging results in treating cancer, most research lacks evidence from clinical trials. This situation arises from several key factors. Firstly, many current TRPM channel modulators lack specificity, not only affecting target molecules but also non-target ones. Secondly, these drugs often have limited efficacy, requiring specific optimization to effectively modulate channel activity in cancer therapy. Finally, TRPM4, TRPM7, TRPM8, and other channels are widely expressed throughout the body, and drugs targeting them are likely to cause systemic side effects in clinical practice (such as targeting TRPM8 channels can easily cause temperature sensation and regulation disorders).

With the advancement of AI-assisted drug design, we hope to use this tool for virtual screening and structural optimization to quickly obtain channel modulators with high specificity and effectiveness, thereby improving efficiency and reducing costs. Effective modulators of TRPM channels based on Antibody-Drug Conjugates (ADCs) or nanoparticle delivery systems should also be considered in the future. These approaches can not only improve the efficacy of tumor therapy but also reduce systemic effects in patients and minimize the generation of drug-related side effects.

Targeted therapy and cancer immunotherapy represent two important strategies in cancer treatment. While targeted therapies are known for their rapidity and precision, patients are prone to develop drug resistance during treatment, limiting their long-term efficacy. In contrast, cancer immunotherapies can offer long-lasting efficacy and broader applicability but may incur immune-related side effects and high costs [187]. Overall, both therapies have their own benefits and drawbacks, and combined treatment is emerging as a trend. Notably, drugs targeting TRPM may be used in conjunction with cancer immunotherapy, but more research is still needed to support this.

## Conclusion and prospective

Currently, cancer treatment is shifting from traditional methods to targeted therapies. Notably, ion channels have emerged as the third most common drug target, following enzymes and receptors. This shift highlights the potential of targeting ion channels for more effective and personalized cancer treatments. Numerous studies published in the last ten years have shown the important role of TRPM channels in tumor progression. Here, we summarize their mechanisms of action in several common cancers. Extensive fundamental research indicates that active regulators of TRPM channels have promising prospects in tumor therapy. However, relevant clinical trials date back more than a decade, revealing some issues in this field. Firstly, drug targeting urgently needs improvement. It is necessary to develop small molecule medications with high specificity to regulate TRPM channel function. We believe that TRPM effective channel modulators utilizing antibody-drug conjugate (ADC) forms or employing nanoparticles for delivery can significantly address the current challenges in treatment. Secondly, many studies remain at the cellular level, which may contribute to ongoing controversies. Future research should focus on animal models and organoid models to better inform clinical experiments. Notably, inhibition of autophagy can enhance the therapeutic effect of many chemotherapeutic drugs and overcome drug resistance. Therefore, inhibiting autophagy in cancer cells has become a key strategy for targeting TRPM channels to treat tumors. In this review, we also discuss the potential role of TRPM channels in cancer immunotherapy, including the possibility of combining TRPM channel modulators with ICIs. We anticipate that this strategy will be the next breakthrough in targeting TRPM channels for the treatment of tumors.

## Data Availability

No datasets were generated or analysed during the current study.

## Abbreviations

ADCs: Antibody-Drug Conjugates
ATGs: Autophagy-related Genes
CaM: Calmodulin
CAMKII: Calcium/calmodulin-dependent protein kinase II
CaMKK2: Calcium/calmodulin-dependent protein kinase kinase 2
ccRCC: Clear Cell Renal Cell Carcinoma
CiSP: Cisplatin
CRC: Colorectal Cancer
ECM: Extracellular Matrix
EMT: Epithelial-Mesenchymal Transition
ICIs: Immune Checkpoint Inhibitors
MMPs: Matrix Metalloproteinases
NFAT: Nuclear Factor of Activated T cells
OS: Overall Survival
PC: Pancreatic Cancer
PCa: Prostate Cancer
PDAC: Pancreatic Ductal Adenocarcinomas
RNS: Reactive Nitrogen Species
ROS: Reactive Oxygen Species
TME: Tumor Microenvironment
TRP: Transient Receptor Potential
TRPM: Transient Receptor Potential Melastatin
TCAF2: TRP Channel-Associated Factor 2
